# 30-Year High Cardiovascular Risk Incidence and its Determinants: CUME Study

**DOI:** 10.1590/0034-7167-2022-0544

**Published:** 2023-12-04

**Authors:** Renata Soares Passinho, Josefina Bressan, Helen Hermana Miranda Hermsdorff, Fernando Luiz Pereira de Oliveira, Adriano Marçal Pimenta

**Affiliations:** I Universidade Federal de Minas Gerais. Belo Horizonte, Minas Gerais, Brazil; II Universidade Federal de Viçosa. Viçosa, Minas Gerais, Brazil; III Universidade Federal de Ouro Preto. Ouro Preto, Minas Gerais, Brazil; IV Universidade Federal do Paraná. Curitiba, Paraná, Brazil

**Keywords:** Incidence, Cardiovascular Risk, Risk Factors, Cohort Studies, Epidemiologic Determinants, Incidencia, Riesgo Cardiovascular, Factores de Riesgo, Estudios de Cohortes, Determinantes Epidemiológicos, Incidência, Risco Cardiovascular, Fatores de Risco, Estudos de Coortes, Determinantes Epidemiológicos

## Abstract

**Objective:**

Estimate the incidence of the 30-year high cardiovascular risk and its determinants among graduates of federal universities in Minas Gerais.

**Methods:**

This is a prospective cohort of 2,854 adults aged 20 to 59. The incidence of the outcome was calculated using the Framingham equation and its determinants were determined through multivariate Cox regression.

**Results:**

After an average of 2.62 years, the incidence of high cardiovascular risk was 8.09 and 20.1 cases per 1,000 person-years, for females and males respectively. Being male (HR: 2.34; 95% CI: 1.58 - 3.46), employment (HR: 2.13; 95% CI: 1.13 - 3.99), high consumption of processed foods (HR: 2.44; 95% CI: 1.21 - 4.90), and being physically active (HR: 0.63; 95% CI: 0.41 - 0.98) were associated with high cardiovascular risk.

**Conclusions:**

Among highly educated adults, being male, employment, and high consumption of processed foods are predictors of high cardiovascular risk, while being physically active acts as a protective factor.

## RESUMO

**Objetivo:**

Estimar a incidência do alto risco cardiovascular de 30 anos e seus determinantes em egressos de universidades federais mineiras**. Métodos:** Coorte prospectiva com 2.854 adultos de 20 a 59 anos. Calculou-se a incidência do desfecho pela equação de Framingham e seus determinantes pela regressão de Cox multivariada.

**Resultados:**

Após uma média de 2,62 anos, a incidência do alto risco cardiovascular foi de 8,09 e 20,1 casos/1.000 pessoas-ano, respectivamente, nos sexos feminino e masculino. O sexo masculino (HR: 2,34; IC95%: 1,58 - 3,46), trabalhar (HR: 2,13; IC95%: 1,13 - 3,99), elevado consumo de alimentos processados (HR: 2,44; IC95%: 1,21 - 4,90) e ser ativo fisicamente (HR: 0,63; IC95%: 0,41 - 0,98) associaram-se ao alto risco cardiovascular.

**Conclusões:**

Em adultos com alta escolaridade, o sexo masculino, trabalhar e o elevado consumo de alimentos processados são preditivos do alto risco cardiovascular, enquanto ser ativo fisicamente é um fator de proteção.

## RESUMEN

**Objetivo:**

Estimar la incidencia del alto riesgo cardiovascular de 30 años y sus determinantes en graduados de universidades federales de Minas Gerais.

**Métodos:**

Cohorte prospectiva con 2.854 adultos de 20 a 59 años. Se calculó la incidencia del desenlace usando la ecuación de Framingham y sus determinantes a través de la regresión multivariante de Cox.

**Resultados:**

Después de un promedio de 2,62 años, la incidencia del alto riesgo cardiovascular fue de 8,09 y 20,1 casos por 1.000 personas-año, respectivamente, en los géneros femenino y masculino. Ser masculino (HR: 2,34; IC95%: 1,58 - 3,46), trabajar (HR: 2,13; IC95%: 1,13 - 3,99), un alto consumo de alimentos procesados (HR: 2,44; IC95%: 1,21 - 4,90) y ser activo físicamente (HR: 0,63; IC95%: 0,41 - 0,98) se asociaron con el alto riesgo cardiovascular.

**Conclusiones:**

En adultos con alta educación, ser masculino, trabajar y el alto consumo de alimentos procesados son predictores del alto riesgo cardiovascular, mientras que ser activo físicamente es un factor de protección.

## INTRODUCTION

Cardiovascular diseases pose significant social and economic burdens in Brazil and around the world, as they are among the leading causes of death and physical disability in working-age adults^(1)^. The primary prevention of these diseases involves lifestyle changes, as adopting various healthy habits has a synergistic effect greater than that of one specific habit alone^(2)^. To assist with lifestyle changes, multivariable risk prediction algorithms have been developed to aid in the assessment of cardiovascular risk^(3)^. From this perspective, several equations evaluate cardiovascular risk; the most widely used is the Framingham score^(4)^.

The Framingham score can predict the risk of cardiovascular disease over 10^(4)^ or 30 years^(3)^. The 10-year score assesses the cardiovascular risk of individuals between the ages of 30 and 74^(4)^ and considers outcomes such as coronary heart disease, stroke, peripheral arterial disease, and heart failure^(3)^. The 30-year score gauges the cardiovascular risk in individuals aged 20 to 59 and accounts for outcomes including coronary death, myocardial infarction, stroke, combined coronary artery disease with coronary insufficiency, and chest pain. It also considers stroke associated with transient ischemic attack, intermittent claudication, and congestive heart failure^(3)^. In the 10-year score, a cardiovascular risk greater than 20% is deemed high^(4)^, whereas in the 30-year score, a risk exceeding 40% is classified as high^(3)^.

There are two equations to calculate the 10 or 30-year cardiovascular risk. One uses non-laboratory variables easily obtained during primary health care (age; body mass index - BMI; systolic blood pressure - SBP; use of antihypertensive medication; current smoking status; and diabetes diagnosis), and the other substitutes BMI with laboratory variables (total cholesterol and high-density lipoprotein cholesterol - HDL-c). Studies on the Framingham score have demonstrated effectiveness when using non-laboratory variables in their cardiovascular risk prediction models^(3-4)^.

Most studies on the topic utilize the 10-year score^(5-7)^, primarily because it was the inaugural global tool to precisely estimate the comprehensive risk of cardiovascular disease and its individual determinants. It also streamlined risk prediction in clinics by replacing disease-specific algorithms with a single global tool for predicting cardiovascular diseases. Nonetheless, it tends to underestimate cardiovascular risk in younger individuals and women^(4)^, potentially discouraging the maintenance of healthy habits or adherence to treatments that prevent future cardiovascular diseases^(8)^.

To date, factors associated with the 30-year cardiovascular risk have been explored in a limited number of studies, including a non-randomized controlled clinical trial^(9)^ and a cross-sectional study^(10)^. It is vital to longitudinally assess the 30-year cardiovascular risk to comprehend its determinants and suggest or refine preventive strategies. This perspective gains significance when considering the sample of this study: even being educated young adults, they exhibit a high prevalence of cardiovascular risk factors^(11)^. Occupying prominent societal positions, the cardiovascular morbidity and mortality of this group might further exacerbate the social and economic challenges faced by the country.

The Framingham score was conceived with an emphasis on Primary Health Care (PHC), chiefly because it facilitates the use of non-laboratory variables (replacing plasma concentrations of total cholesterol and HDL-c with BMI) in one of its equations^(3-4)^. Consequently, it provides a cardiovascular risk estimate at a more economical cost to the health system.

In Brazil, nurses, integral to the multi-professional PHC team, play pivotal roles in the educational and care activities catered to health service users, particularly those with non-communicable chronic diseases^(12)^. Employing the 30-year Framingham score in tandem with knowledge about high cardiovascular risk determinants augments the efficacy of these professionals. The emphasis is on promoting the reduction of risk factors and bolstering protective factors in young adults, thereby diminishing the likelihood of future cardiovascular incidents and enhancing the long-term prognosis for those who have previously experienced a cardiovascular event.

## OBJECTIVE

To estimate the 30-year incidence of high cardiovascular risk and its determining factors among graduates from federal universities in Minas Gerais.

## METHODS

### Ethical Aspects

The Minas Gerais University Cohort (CUME Study) is conducted in accordance with the ethical guidelines set forth by resolutions no. 466/2012 and no. 510/2016 of the National Health Council. It received approval from the Research Ethics Committee of the Federal University of Minas Gerais. All participants in the study have read and accepted the online Informed Consent Form.

### Design, Period, and Location of the Study

This research is a sub-project of the CUME study, an open and observational epidemiological cohort study that has been ongoing in Brazil since 2016. It involves alumni from seven universities in the state of Minas Gerais, including the Federal University of Minas Gerais, Federal University of Viçosa, Federal University of Ouro Preto, Federal University of Lavras, Federal University of Juiz de Fora, Federal University of Alfenas, and Federal University of the Jequitinhonha and Mucuri Valleys. The study aims to assess the impact of the Brazilian population’s dietary patterns and the nutritional transition on non-communicable chronic diseases. The recruitment of participants is ongoing, allowing for continuous growth in the sample size with each biennial follow-up wave. Previously recruited participants receive updated questionnaires (Q_2, Q_4, ..., Q_n), while new participants are given the baseline questionnaire (Q_0). Further details can be found in previous publications^(11,13-16)^. As the CUME Study is multicentric in nature, there might be similarities in terms of variable definition, description, categorization, and data collection across its various publications. Moreover, the Strengthening the Reporting of Observational Studies in Epidemiology (STROBE) guideline was employed to shape this article’s methodology.

### Sample, Inclusion, and Exclusion Criteria

Participants are alumni of the aforementioned universities, 18 years of age or older, and of either gender. The baseline data from 2016 and the two subsequent follow-up waves (from 2018 and 2020) of the CUME Study were used for this research. Out of the 4,057 participants who responded to the baseline questionnaire, several were excluded for various reasons: 40 were foreigners; 289 were Brazilian residents living abroad; 275 were women who were either pregnant or within one year postpartum; six had an energy intake of less than 500 kcal^(14)^; and 208 had an energy intake of 6000 kcal^(14)^ or more. This left a sample of 3,239 participants. From this group, individuals who did not meet the criteria for the 30-year global cardiovascular risk^(3)^ calculations were further excluded: those younger than 20 years (n = 2) or older than 59 (n = 85); those with a prevalent cardiovascular disease (n = 106); those diagnosed with cancer (n = 60); or those presenting a high 30-year global cardiovascular risk at the study’s inception (n = 132). The final sample size stood at 2,854 participants.

### Study Protocol

Data collection for the baseline of the cohort was conducted by having participants complete the online project questionnaire (Q_0). This questionnaire was divided into two sections: the first addressed socioeconomic variables, lifestyle, self-reported morbidity, current medications, a history of clinical and laboratory tests conducted in the past two years, and anthropometric measures. The second section, known as the Food Frequency Questionnaire (FFQ), listed 144 food items, organized into eight groups: dairy, meats and fish, cereals and legumes, oils and fats, fruits and vegetables, beverages, and other foods and food preparations.

For the two follow-up waves, data were collected through the online questionnaires at the two (Q_2) and four (Q_4) year intervals. Q_2 covered variables related to daily living activities, such as autonomy in personal hygiene, basic human needs, and nutrition; pregnancies after Q_0; current weight; results from clinical and laboratory tests; ongoing medications; lifestyle; and disease diagnoses during the study’s follow-up period. Q_4 assessed variables related to employment status, current weight, results from clinical and laboratory tests, disease diagnoses during the follow-up, continuous medication use, lifestyle, and sleep patterns.

### Outcome Variable: 30-year High Cardiovascular Risk

In the current study, the Framingham equation was chosen to calculate the 30-year cardiovascular risk. This equation incorporates BMI as a substitute for plasma concentrations of total cholesterol and HDL-c^(3)^. This choice stemmed from a previous study^(11)^ with a sub-sample from CUME. In this study, self-reported data on weight, height, and BMI closely matched measurements taken directly by the researchers. This is evidenced by the high Intraclass Correlation Coefficients (ICC) of 0.989, 0.995, and 0.983, respectively. Conversely, self-reported data on total cholesterol were not validated. However, data on HDL-c showed substantial agreement with researcher measurements (ICC = 0.761).

Participants, in both baseline and follow-up questionnaires, provided data integral to the equations for calculating cardiovascular risk. These included: weight, height (from which BMI was derived), gender, age, current smoking status (yes or no), self-reported systolic blood pressure, use of antihypertensive medication (yes or no), a medical diagnosis of type 2 diabetes mellitus (yes or no), and hypertension (yes or no). Cardiovascular risk was thus computed at the baseline and during two subsequent follow-up periods. It was then categorized into low risk (< 12%), intermediate risk (≥ 12% and < 40%), or high risk (≥ 40%)^(3,10)^. The incidence of a high cardiovascular risk was determined based on participants who, being initially risk-free at baseline, were later categorized as high risk during the follow-up.

### Covariates: Determinants of 30-year High Cardiovascular Risk

Covariates were sourced from the baseline questionnaires and categorized per the standards of the CUME Study, which have been outlined in previous research^(11,13-16)^. These covariates encompassed: marital status, race/ethnicity, academic field, employment status, family income, and lifestyle factors like smoking habits, alcohol consumption patterns, physical activity levels, and dietary habits. The health conditions evaluated included the systolic blood pressure value, usage of antihypertensive medication, diagnosis of type 2 diabetes mellitus (DM2), and BMI values.

Family income, presented as a continuous variable, was gauged in terms of minimum wages and on a per capita basis. It adhered to the prevalent value of the year when participant data was gathered. Heavy episodic alcohol consumption, a metric for alcoholism, was delineated as the intake of four or more alcoholic drinks for women and five or more for men on any single occasion within the past 30 days^(17)^. This consumption pattern was initially bifurcated into “yes” or “no.” Affirmative respondents were further probed on the frequency of such consumption within a month.

Physical activity levels were determined by a compilation of 24 leisure activities, itemized in terms of minutes spent per week^(18)^. This data was initially classified by intensity - light, moderate, and vigorous. Later, a variable termed “level of physical activity” was introduced, segmenting activities into “active,” “insufficiently active,” or “inactive”^(18)^.

The food frequency questionnaire offered insights into each participant’s dietary preferences. After selecting a food item, participants were required to specify portion sizes using common household measures. The data, initially captured in weekly, monthly, and yearly frequencies, was then distilled into daily consumption metrics. This data was cross-referenced with authoritative food composition tables from Brazil^(19-20)^ and the U.S. Department of Agriculture^(21)^.

The food items from the questionnaire were cataloged based on the degree and intent of industrial processing, adhering to the NOVA Classification system. In this analysis, unprocessed and minimally processed foods were combined with cooking ingredients due to their typical consumption patterns^(22)^. The caloric contribution by processing level was deduced by aggregating the caloric values across food groups, normalized by total energy intake. These derived variables were then segmented into quintiles, using the lowest quintile as the reference benchmark for subsequent analysis. The reliability of the food frequency questionnaire’s self-reported data was previously validated in a CUME study^(14)^. In it, the questionnaire displayed a moderate agreement when juxtaposed against a 24-hour food recall, especially for specific food groups. However, the correlation was slightly weaker for unprocessed or minimally processed foods and cooking ingredients, albeit still nearing the threshold for moderate agreement^(23)^.

### Analysis of Results and Statistics

The variable “systolic blood pressure” had more than 10% missing data. At baseline, we had data for 504 individuals, 738 at the two-year follow-up, and 1591 at the four-year follow-up. Due to these missing data, we implemented the process of multiple imputation^(24)^. The baseline characteristics of participants-pertaining to demographic, socioeconomic, lifestyle, dietary intake, and components of the Framingham score-were outlined based on the incidence of high cardiovascular risk, presenting both absolute and relative frequencies. Statistical differences were assessed using either Pearson’s chi-squared test or the t-test, with a statistical significance level set at 5%.

The follow-up duration was calculated in person-years for each participant. This was determined by the difference between the date the follow-up questionnaire (where the high cardiovascular risk was identified) was completed and the date the baseline questionnaire was completed. If the outcome was not identified, the difference was computed between the date of the last follow-up questionnaire and the date of the baseline questionnaire.

A hierarchical multivariate analysis was performed using the Cox regression technique. This method was used to derive the proportional hazard ratios (Hazard Ratio - HR)^(25)^, where the dependent variable was the time leading up to the event’s occurrence, and participants were represented as person-time^(25-26)^. For this study’s multivariate analysis, variables were categorized into two blocks, in line with previously proposed theoretical models^(27-28)^:

1. Distal block: demographic and socioeconomic variables;
2. Proximal block: lifestyle and dietary habits, categorized by their degree of processing.

Initially, variables that had a 20% association level with the outcomes in the bivariate analysis were chosen for the final model. Following that, every variable from the distal block was incorporated into the final model based on descending statistical significance. They were then iteratively removed using the backward method until only those with a statistical significance level below 5% remained. This procedure was repeated for the proximal block variables. Consequently, by the end of this process, the variables from the initial block were adjusted for those in the subsequent block.

## RESULTS

The study encompassed a total of 2,854 participants (864 males and 1,990 females). After an average follow-up period of 2.62 years (standard deviation = 0.96 years), 101 participants developed high cardiovascular risk: 54 within two years and 47 within four years of follow-up. As such, the incidence of high cardiovascular risk was 8.09 cases/1000 person-years among female participants and 20.1 cases/1000 person-years among male participants. Moreover, the 101 participants who displayed an incidence of the outcome had an average cardiovascular risk of 30.05% at baseline (SD = 6.99%), with a median of 31.67%, and interquartile ranges spanning from 25.69% to 35.46%. Out of these, 94 fell into the medium-risk category, while seven were categorized as low cardiovascular risk.

In comparison to participants without a high cardiovascular risk, those diagnosed with the condition were more likely to be male, have a higher average age, be in stable relationships, have undergone specialized education, be employed, smoke, engage in heavy episodic drinking (≥ 5 times/month), and consume processed foods. The health conditions of participants with a high cardiovascular risk were notably poorer, as evidenced by higher systolic blood pressure, more frequent reliance on antihypertensive drugs, a greater prevalence of type 2 diabetes mellitus, and an elevated BMI (Table 1).

**Table 1:** Baseline characteristics of 2,854 participants according to the diagnosis of high cardiovascular risk at age 30: CUME Study, 2016-2020. Belo Horizonte, Minas Gerais, Brazil, 2022

Characteristics		High Cardiovascular Risk	
		No (n = 2,753) n (%)	Yes (n = 101) n (%)
Demographic and Socioeconomic			
Gender ^*^			
Female		1,941 (70.50)	49 (48.51)
Male		812 (29.50)	52 (51.49)
Age [average, (SD)]^†^		34.16 (7.56)	44.25 (7.27)
Race/Skin Color			
White		1,765 (64.11)	68 (67.33)
Black/Brown/Yellow/Indigenous		988 (35.89)	33 (32.67)
In a Stable Relationship ^*^			
No		1,478 (53.69)	42 (41.58)
Yes		1,275 (46.31)	59 (58.42)
Healthcare Professional			
No		1,973 (71.67)	78 (77.23)
Yes		780 (28.33)	23 (22.77)
Education Level ^*^			
Undergraduate		741 (26.92)	20 (19.80)
Specialization		619 (22.48)	34 (33.66)
Master's/Doctorate		1,393 (50.60)	47 (46.53)
Currently Employed ^*^			
No		678 (24.63)	11 (10.89)
Yes		2,075 (75.37)	90 (89.11)
Family Income (minimum wages) [average (SD)]		10.38 (18.23)	11.93 (7.56)
Lifestyle Habits			
Current Smoking ^*^			
No		2,551 (92.66)	86 (85.15)
Yes		202 (7.34)	15 (14.85)
Heavy Episodic Drinking (times/month)^*^			
0		1,604 (58.26)	62 (61.39)
1 to 2		617 (22.41)	14 (13.86)
3 to 4		313 (11.37)	9 (8.91)
5 or more		219 (7.95)	16 (15.84)
Physical Activity (min/week)			
Sedentary		635 (23.07)	32 (31.68)
Insufficient		574 (20.85)	16 (15.84)
Active		1,544 (56.08)	53 (52.48)
Dietary Intake (grams/day)			
Natural Foods/Culinary Ingredients [average (SD)]		65.98 (13.29)	66.30 (11.43)
Processed Foods [average (SD)]^†^		9.90 (5.79)	11.47 (6.80)
Ultraprocessed Foods [average (SD)]		25.31 (11.17)	23.99 (10.50)
Health Conditions			
Systolic Blood Pressure (mmHg) [average (SD)]^†^		113.68 (10.26)	120.40 (11.60)
Use of Antihypertensive Medication ^*^			
No		2,667 (96.88)	74 (73.27)
Yes		86 (3.12)	27 (26.73)
Diagnosis of Type 2 Diabetes Mellitus ^*^			
No		2,725 (98.98)	91 (90.10)
Yes		28 (1.02)	10 (9.90)
Body Mass Index (BMI) (Kg/m^2) [average (SD)]^†^		24.15 (4.05)	29.00 (4.88)

Note: SD = standard deviation;

* p value < 0.05 by Pearson's chi-squared test; †p value < 0.05 by t-Student test.

Regarding the demographic and socioeconomic variables, being male, having completed a specialization, and being employed were associated with high cardiovascular risk (Table 2).

**Table 2:** Demographic and socioeconomic factors associated with high cardiovascular risk at 30 years in 2,854 participants. CUME Study, 2016-2020. Belo Horizonte, Minas Gerais, Brazil, 2022

Characteristics	Incidence (cases/1.000 person-years)	HR^*^	95% CI^*^
Demographic and Socioeconomic			
Gender			
Female	8.09	1 (Ref.)	-
Male	20.10	2.42	1.64 - 3.58
Race/Skin Color			
White	12.25	1 (Ref.)	-
Black/Brown/Yellow/Indigenous	10.67	0.85	0.56 - 1.29
Stable Relationship			
No	14.37	1 (Ref.)	-
Yes	9.25	1.43	0.96 - 2.13
Healthcare Professional			
No	12.61	1 (Ref.)	-
Yes	9.37	1.29	0.81 - 2.06
Education Level			
Bachelor's Degree	8.92	1 (Ref.)	-
Specialization	17.25	1.89	1.09 - 3.29
Master's/Doctorate	10.61	1.10	0.65 - 1.87
Employed			
No	5.49	1 (Ref.)	-
Yes	13.55	2.29	1.22 - 4.29
Family Income (in minimum wages)			
< 5	8.36	1 (Ref.)	-
5 to 9	10.26	1.03	0.55 - 1.92
10 and above	13.58	1.45	0.80 - 2.63

*Note: HR = Hazard Ratio; CI = 95% Confidence Interval.*

Regarding lifestyle and dietary habits, excessive alcohol consumption (≥ 5 times/month) and consumption of processed foods were associated with a high cardiovascular risk (Table 3).

**Table 3:** Lifestyle and dietary habits associated with a high cardiovascular risk at 30 years in 2,854 participants. CUME Study, 2016-2020. Belo Horizonte, Minas Gerais, Brazil, 2022

Characteristics	Incidence (cases/1.000 person-years)	HR^*^	95% CI^*^
Lifestyle Habits			
Physical Activity			
Sedentary	15.89	1 (Ref.)	-
Insufficient	8.81	0.57	0.31 - 1.05
Active	11.01	0.68	0.44 - 1.06
Heavy Episodic Drinking (times/month)			
0	12.29	1 (Ref.)	-
1 to 2	7.25	0.56	0.31 - 1.00
3 to 4	9.22	0.74	0.36 - 1.49
5 or more	23.09	1.98	1.14 - 3.44
Dietary Habits			
Whole Foods/Culinary Ingredients			
1st quintile	11.18	1 (Ref.)	-
2nd quintile	11.86	1.01	0.55 - 1.87
3rd quintile	8.65	0.74	0.38 - 1.45
4th quintile	14.15	1.23	0.68 - 2.24
5th quintile	12.69	1.12	0.61 - 2.08
Processed Foods			
1st quintile	6.28	1 (Ref.)	-
2nd quintile	12.93	2.07	1.01 - 4.25
3rd quintile	9.27	1.38	0.64 - 2.99
4th quintile	13.01	2.14	1.04 - 4.42
5th quintile	17.11	2.81	1.40 - 5.63
Ultra-Processed Foods			
1st quintile	13.68	1 (Ref.)	-
2nd quintile	12.90	0.91	0.50 - 1.64
3rd quintile	12.87	0.93	0.51 - 1.69
4th quintile	9.94	0.70	0.38 - 1.32
5th quintile	9.39	0.65	0.34 - 1.23

*Note: HR = Hazard Ratio; CI = 95% Confidence Interval.*

After performing the hierarchical multivariate analysis using the Cox regression technique, the 30-year high cardiovascular risk was positively associated with being male (HR: 2.34; CI 95%: 1.58 - 3.46), being employed (HR: 2.13; CI 95%: 1.13 - 3.99), and high consumption of processed foods (HR: 2.44; CI 95%: 1.21 - 4.90). It was negatively associated with engaging in 150 minutes or more of physical activity per week (HR: 0.63; CI 95%: 0.41 - 0.98) (Table 4).

**Table 4:** Determinants of the 30-year high cardiovascular risk in 2,854 participants. CUME Study, 2016-2020. Belo Horizonte, Minas Gerais, Brazil, 2022

Characteristics	HR^*^	95% CI^*^
Distal Block		
Demographic and Socioeconomic		
Gender		
Female	1 (Ref.)	-
Male	2.34	1.58 - 3.46
Employed		
No	1 (Ref.)	-
Yes	2.13	1.13 - 3.99
Proximal Block		
Lifestyle Habits		
Physical Activity (min/week)		
Sedentary	1 (Ref.)	-
Insufficient	0.59	0.32 - 1.07
Active	0.63	0.41 - 0.98
Dietary Habits		
Processed Foods		
1st quintile	1 (Ref.)	-
2nd quintile	2.01	0.97 - 4.12
3rd quintile	1.23	0.57 - 2.66
4th quintile	1.84	0.89 - 3.82
5th quintile	2.44	1.21 - 4.90

*Note: HR = Hazard Ratio; CI = 95% Confidence Interval.*

## DISCUSSION

This study aimed to estimate the 30-year high cardiovascular risk incidence and its determinants among CUME participants. After an average follow-up of 2.62 years (standard deviation = 0.96 years), an incidence of 8.09 cases/1,000 person-years was observed for females and 20.1 cases/1,000 person-years for males. Furthermore, being male, being employed, and consuming processed foods were positively associated with a 30-year high cardiovascular risk, while engaging in physical activity for 150 minutes or more per week was negatively associated.

This research is groundbreaking because, after an extensive literature review, it was the first to study the 30-year high cardiovascular risk incidence. A previous study on the Mexican population analyzed the 10-year risk^(29)^. In that study, the threshold set for high cardiovascular risk was 10%, while the researchers in the Framingham study set a 20% threshold for outcome detection^(3)^. Moreover, the Mexican cohort aimed to study the association between dietary patterns and the development of high cardiovascular risk, while this study investigated risk factors related not only to diet but also to sociodemographic characteristics and lifestyle habits. Hence, comparing our scientific findings with others can be challenging due to the uniqueness of the analyzed outcome.

The 30-year score is particularly noteworthy when compared to the 10-year one. It allows for a more comprehensive risk assessment in younger populations and includes additional cardiovascular outcomes, such as angina pectoris, transient ischemic attack, intermittent claudication, and congestive heart failure, not considered in the 10-year score^(3)^.

In this study, the 30-year high cardiovascular risk incidence was higher among male participants. This finding is supported by a cross-sectional study in Suriname that investigated the 10-year high cardiovascular risk, modified for Africans (≥ 10%) and Asians (≥ 12%). The outcome was observed in 19.7% of participants, with higher proportions in men than in women for both ethnic groups^(30)^. Another cross-sectional study that examined the 30-year cardiovascular risk among 352 homeless individuals with mental illness indicated that men were more likely to have an intermediate or high risk^(10)^. However, both aforementioned studies are cross-sectional and may be subject to reverse causality.

Cohort studies focusing on cardiovascular disease incidence outcomes also align with our findings. A Dutch cohort^(31)^ revealed a higher 30-year incidence of myocardial infarction among men. An English cohort^(32)^ indicated that the incidence of cardiovascular diseases was higher in men across all age groups. Conversely, a Dutch cohort study that tracked 8,419 participants (60.9% women) aged ≥ 55 years without prior diseases over 20.1 years found that, at age 55, the lifetime risk of developing any cardiovascular disease was similar for both men and women^(33)^. Therefore, due to estrogenic protection in the myocardium during the reproductive period, women have a lower risk of developing cardiovascular diseases^(34)^.

In this study, employment was associated with an increased 30-year cardiovascular risk. This finding aligns with a Brazilian cross-sectional study involving 211 workers of both genders. The study investigated the association between night shifts and high cardiovascular risk using the 10-year Framingham score, concluding that night shifts increased the prevalence of high cardiovascular risk by 67%^(35)^. Our longitudinal findings can be compared to a systematic review of 42 cohort studies, encompassing data from 603,838 participants. The review demonstrated that working long hours (≥ 55 hours per week) elevated the risk of coronary disease by 1.13 times^(36)^. The adverse effects of extended work hours on cardiovascular health can be attributed to reduced time for activities outside of work and heightened exposure to psychosocial, physical, and chemical occupational hazards^(36)^. Furthermore, the pathophysiology of stress encompasses autonomic, metabolic, inflammatory, and hemostatic disorders, potentially leading to the formation or rupture of atherosclerotic plaques^(37)^.

In the current study, it was demonstrated that being physically active reduces the incidence of high cardiovascular risk over 30 years. This finding is supported by the results of a cross-sectional study involving 265 Nigerians living with the human immunodeficiency virus (HIV). The study highlighted that the prevalence of a moderate to high 10-year coronary risk was 11.7%, and that low physical activity was observed in 66% of the participants^(38)^. However, no association analyses were conducted between the traditional risk factors in the sample and the high cardiovascular risk score. Another cross-sectional study, involving 888 individuals from a Spanish province, linked the 10-year cardiovascular risk to metabolic syndrome. It was found that 29.7% of the participants had a high cardiovascular risk, but a high level of physical activity reduced this risk by 60%^(39)^.

A cohort study with 11,351 participants estimated the 30-year cardiovascular risk and revealed that individuals who increased their physical activity levels had a significantly lower risk of heart failure. In contrast, those who decreased their activity experienced an increased risk^(40)^. Regular physical activity decreases the risk of cardiovascular disease^(40)^, primarily due to the increased expression of antioxidant enzymes in the heart^(41)^. This is crucial, as reactive oxygen species can trigger a series of pathophysiological events leading to cardiomyocyte dysfunction, cellular apoptosis, contractile dysfunction, impaired cardiac remodeling, fibrosis, hypertrophy, and heart failure^(42)^.

Regarding dietary consumption, this study observed that a higher intake of processed foods increased the incidence of a high 30-year cardiovascular risk. Food processing involves physical, biological, and chemical procedures applied after foods are harvested and before they are consumed or prepared into dishes and meals. Examples include canned vegetables and fish, fruits in syrup, cheeses, and bread. These processes often involve the addition of salt, oil, sugar, or other substances, aiming to extend shelf life or enhance the sensory qualities of fresh or minimally processed foods^(22)^. However, while the basic identity and most nutrients of the original food are preserved, the nutritional composition is unfavorably altered due to processing^(43)^. As a result, processed foods contain increased amounts of oil, salt, and sugar, and excessive consumption of these ingredients is a dietary risk factor for cardiovascular diseases^(44)^.

Most participants who experienced a high cardiovascular risk incidence were in the intermediate risk category at the start of the study (n = 94). Yet, seven transitioned from the low-risk category to the high-risk category. Both groups could benefit from health promotion and disease prevention measures, as these have the potential to modify certain components of the 30-year cardiovascular risk score, such as smoking, overweight, and systolic blood pressure, thereby reducing the high cardiovascular risk over 30 years.

### Study limitations

The limitations of this study include self-reporting of the variables that make up the Framingham score for 30-year global cardiovascular risk and the exposure variables. However, it is important to note that the self-reported variables that are components of the Framingham score^(11)^ and some exposure variables, particularly dietary variables^(14)^, have been validated in previous studies. Additionally, in general, self-reported data from participants with high education have good accuracy^(44)^. It is important to note that, even after multivariate adjustment, there may be residual confounding, as high cardiovascular risk among young adults may be due to exposure to other determinants not evaluated in this study, such as the use of illicit substances^(10)^, heredity^(45)^ and infectious diseases, such as COVID-19^(46)^.

### Contributions to the field of Nursing, Health or Public Policy

To stimulate behaviors related to the reduction of high cardiovascular risk, it becomes relevant to include, during the collection of data from Nursing and other health professionals with the user, questions that go beyond the traditional cardiovascular risk factors that make up the Framingham score. Factors such as lifestyle and dietary intake are essential, especially in the context of Primary Health Care, whose services prioritize the planning and implementation of public actions for the prevention and control of risk factors and cardiovascular diseases.

## CONCLUSIONS

Our results suggest that, in adults aged 20 to 59 years with high education, being physically active is a protective factor against the incidence of 30-year high cardiovascular risk. On the other hand, being male, having a job and consuming excessive processed foods increase the chance of the outcome. The identification of determinants that raise 30-year high cardiovascular risk in young adults indicates that current approaches to cardiovascular health promotion may not be adequately reaching this population. Thus, the use of the 30-year Framingham score, as opposed to the 10-year score that is already commonly used, can help in the early screening of modifiable cardiovascular risk factors, promoting beneficial lifestyle changes.
